# Changes in Health Care and Prescription Medication Affordability in the US During the COVID-19 Pandemic

**DOI:** 10.1001/jamahealthforum.2024.1939

**Published:** 2024-06-30

**Authors:** Stephen A. Mein, Lucas X. Marinacci, ZhaoNian Zheng, Isabella Ahmad, Rishi K. Wadhera

**Affiliations:** 1 Richard A. and Susan F. Smith Center for Outcomes Research, Beth Israel Deaconess Medical Center, Boston, Massachusetts; 2 Harvard Medical School, Boston, Massachusetts

## Abstract

**Importance:**

In the US, the COVID-19 pandemic led to a significant rise in unemployment and economic loss that disproportionately impacted low-income individuals. It is unknown how health care and prescription medication affordability changed among low-income adults during the COVID-19 pandemic overall and compared with their higher-income counterparts.

**Objective:**

To evaluate changes in health care affordability and prescription medication affordability during the COVID-19 pandemic (2021 and 2022) compared with pre–COVID-19 pandemic levels (2019) and whether income-based inequities changed.

**Design, Setting, and Participants:**

This retrospective cross-sectional study included adults 18 years and older participating in the National Health Interview Survey (NHIS) in 2019, 2021, and 2022. Low-income adults were defined as having a household income of 200% or less of the federal poverty level (FPL); middle-income adults, 201% to 400% of the FPL; and high-income adults, more than 400% of the FPL. Data were analyzed from June to November 2023.

**Main Outcomes and Measures:**

Measures of health care affordability and prescription medication affordability.

**Results:**

The study population included 89 130 US adults. Among the weighted population, 51.6% (95% CI, 51.2-52.0) were female, and the mean (SD) age was 48.0 (0.12) years. Compared with prepandemic levels, during the COVID-19 pandemic, low-income adults were less likely to delay medical care (2022: 11.2%; 95% CI, 10.3-12.1; 2019: 15.4%; 95% CI, 14.3-16.4; adjusted relative risk [aRR], 0.73; 95% CI, 0.66-0.81) or avoid care (2022: 10.7%; 95% CI, 9.7-11.6; 2019: 14.9%; 95% CI, 13.8-15.9; aRR, 0.72; 95% CI, 0.64-0.80) due to cost, while high-income adults experienced no change, resulting in a significant improvement in income-based disparities. Low-income and high-income adults were less likely to experience problems paying medical bills but experienced no change in worrying about medical bills during the COVID-19 pandemic compared with prepandemic levels. Across measures of prescription medication affordability, low-income adults were less likely to delay medications (2022: 9.4%; 95% CI, 8.4-10.4; 2019: 12.7%; 95% CI, 11.6-13.9; aRR, 0.74; 95% CI, 0.65-0.84), not fill medications (2022: 8.9%; 95% CI, 8.1-9.8; 2019: 12.0%; 95% CI, 11.1-12.9; aRR, 0.75; 95% CI, 0.66-0.83), skip medications (2022: 6.7%; 95% CI, 5.9-7.6; 2019: 10.1%; 95% CI, 9.1-11.1; aRR, 0.67; 95% CI, 0.57-0.77), or take less medications (2022: 7.3%; 95% CI, 6.4-8.1; 2019: 11.2%; 95% CI, 10.%-12.2; aRR, 0.65; 95% CI, 0.56-0.74) due to costs, and these patterns were largely similar among high-income adults. Improvements in measures of health care and prescription medication affordability persisted even after accounting for changes in health insurance coverage and health care use. These patterns were similar when comparing measures of affordability in 2021 with 2019.

**Conclusions and Relevance:**

Health care affordability improved for low-income adults during the COVID-19 pandemic, resulting in a narrowing of income-based disparities, while prescription medication affordability improved for all income groups. These findings suggest that the recent unwinding of COVID-19 pandemic–related safety-net policies may worsen health care affordability and widen existing income-based inequities.

## Key Points

**Question:**

Did health care affordability and prescription medication affordability change for low-income adults during the COVID-19 pandemic, both overall and compared with their higher-income counterparts?

**Findings:**

In this cross-sectional study of 89 130 US adults, measures of health care affordability improved for low-income adults during the COVID-19 pandemic (2021 and 2022) compared with pre–COVID-19 pandemic (2019) levels but largely remained unchanged for high-income adults, which narrowed income-based inequities in health care affordability. In addition, prescription medication affordability improved for both low-income and higher-income adults.

**Meaning:**

These findings suggest that safety-net policies enacted during the COVID-19 pandemic may have prevented health care affordability from worsening, despite increases in unemployment and economic loss, and have important implications as many of these policies come to an end.

## Teaser

This cross-sectional study evaluates changes in health care affordability and prescription medication affordability during the COVID-19 pandemic (2021 and 2022) compared with pre–COVID-19 pandemic levels (2019) and whether income-based inequities changed.

## Introduction

The onset of the COVID-19 pandemic in the US led to a dramatic rise in unemployment, which disproportionately impacted low-income adults.^1,2,3^ As a result, millions of people experienced economic loss, deepening financial strain, and disruptions in health insurance coverage.^4,5^ These changes threatened to worsen low-income adults’ ability to afford medical care and prescription drugs and, ultimately, widen income-based inequities in access to care and health.^6,7,8^

In response, the federal government enacted a series of policies to counteract the negative economic repercussions of the COVID-19 pandemic. Policies like the Families First Coronavirus Response Act, the Coronavirus Aid, Relief, and Economic Security (CARES) Act, and the American Rescue Plan Act provided economic impact funds and enabled many low-income families to maintain Medicaid insurance irrespective of changes in their eligibility.^8,9,10,11^ Although these policy actions helped offset COVID-19 pandemic–related economic and insurance coverage losses for displaced workers during the early phase of the pandemic,^8,12,13^ little is known about whether they prevented health care and prescription medication affordability from worsening as the COVID-19 pandemic went on. On one hand, it is possible that affordability of medical care worsened over the course of the COVID-19 pandemic due to initial loss of income and disruptions in insurance coverage. Alternatively, targeted state and federal protections may have helped mitigate a rise in cost-related barriers to health care services. Understanding how health care and prescription medication affordability changed over the course of the COVID-19 pandemic could inform policy during future public health crises and provide critical insights on the potential implications of the recent unwinding of many COVID-19 pandemic–related safety-net policies.

Therefore, in this study, we aimed to answer 3 questions. First, how did measures of health care affordability change for low-income and higher-income adults during the COVID-19 pandemic (2021 and 2022) compared with prepandemic levels (2019)? Second, did these groups experience a change in prescription medication affordability? And third, did income-based disparities across these measures of affordability change over this period?

## Methods

### Study Population

This cross-sectional study included adults participating in the National Health Interview Survey (NHIS), a nationally representative health survey conducted by the US Census Bureau annually.^14^ The survey collects data from civilian, noninstitutionalized populations within the 50 states and the District of Columbia using a geographically clustered sampling technique and face-to-face interviews with households. Our study population included US adults 18 years or older participating in the 2019 NHIS (pre–COVID-19 pandemic) and in the 2021 and 2022 NHIS (during the COVID-19 pandemic). The 2020 NHIS was excluded because survey responses could reflect both periods and because there were disruptions in the in-person surveys during this year.^15^ Patient demographic and socioeconomic characteristics were self-reported. We classified participants as low income, middle income, or high income. Low income was defined as those with a family income of 200% or less of the federal poverty level (FPL); middle income, 201% to 400% of the FPL; and high income, more than 400% of the FPL, consistent with prior studies.^16,17,18,19^ The Institutional Review Board at Beth Israel Deaconess Medical Center deemed this study exempt because it used publicly available, deidentified data. This study followed the Strengthening the Reporting of Observational Studies in Epidemiology (STROBE) reporting guideline.

### Outcomes

We examined measures of health care affordability, including (1) delaying medical care due to costs, (2) not seeking care due to costs, (3) worrying about paying medical bills, (4) problems paying medical bills, and (5) inability to pay current medical bills among those who reported having problems paying medical bills. In addition, we examined measures of prescription medication affordability (among those taking medications), including (1) not filling prescription medications due to cost, (2) delaying filling prescription medications to save money, (3) skipping prescription medication doses to save money, and (4) taking less prescription medications to save money. All outcomes were self-reported and reflect participant experiences within the last year.

### Statistical Analysis

We compared baseline characteristics between adults in 2019 (pre–COVID-19 pandemic), 2021 (during COVID-19 pandemic), and 2022 (during COVID-19 pandemic) using analysis of variance for continuous covariates and χ^2^ tests for categorical covariates. We then fit survey-weighted logistic regression models to compare outcomes in 2021 and 2022 with outcomes in 2019 after adjustment for age and sex. We did not initially adjust for other covariates (health insurance coverage, health care use, or clinical comorbidities) because they potentially mediated associations between the COVID-19 pandemic and health care affordability. Our models included interaction terms for income level and year to evaluate whether there were differential changes in outcomes during the COVID-19 pandemic across income strata (using high-income adults as the reference group). Next, to understand if pandemic-related changes in health insurance coverage and health care use explained changes in measures of affordability, we repeated our main analysis after including these additional covariates in our models (in addition to age and sex). For these analyses, health insurance coverage was defined as a categorical variable (private insurance; Medicare; Medicaid/public insurance; other, which included Children’s Health Insurance Program, military health insurance, state-sponsored health plans, and Medicare-Medicaid dual eligibility; and uninsured), and health care use was assessed based on the number of urgent care visits, emergency department visits and hospitalizations within the last year. We focused on acute care use because it is associated with higher expenditures, which could make health care less affordable and leave fewer financial resources to spend on medications. In addition, to understand how measures of health care and prescription medication affordability changed among working-age adults who may have been more susceptible to economic changes during the COVID-19 pandemic, we repeated our primary analysis after restricting our study population to adults aged 18 to 64 years and those 65 years and older. We then repeated our analysis among US adults 18 years and older.

Complete case analysis was used for all covariates and outcomes, as missingness rates were low (less than 1%). All statistical tests were 2-sided, with *P* < .05 considered significant. No adjustment was performed for multiple testing. Analyses were performed using SAS Enterprise Guide version 7.15 (SAS Institute).

## Results

The unweighted study population included 89 130 total participants, including 31 997 adults in 2019, 29 482 adults in 2021, and 27 651 adults in 2022. After weighting, 51.6% (95% CI, 51.2-52.0) were female, and the mean (SD) age was 48.0 (0.12) years. Baseline characteristics for US adults prior to the COVID-19 pandemic (2019) and during the COVID-19 pandemic (2021 and 2022) after weighting are shown in Table 1, and unweighted characteristics and characteristics by income level are shown in eTables 1 and 2 in Supplement 1. US adults during the COVID-19 pandemic (2021 and 2022) were less likely to be employed or have low income (≤200% FPL) compared with prepandemic levels (2019). Other socioeconomic characteristics and comorbidities were similar across the study period. Low-income and middle-income adults were more likely to be female, unemployed, and self-report Black race and Hispanic ethnicity compared with high-income adults.

**Table 1.** Baseline Characteristics of US Adults in 2019 (Pre–COVID-19 Pandemic) and 2021 and 2022 (During COVID-19 Pandemic)

Characteristic	US population, % (95% CI)			
	2019 (Pre–COVID-19 pandemic)	2021 (During COVID-19 pandemic)	2022 (During COVID-19 pandemic)	*P* value
Weighted population size, No.^a^	250 899 150	253 141 348	255 344 703	NA
Age, mean (SD), y	47.7 (0.16)	48.1 (0.17)	48.1 (0.16)	.04
Sex				
Female	51.7 (51.0-52.4)	51.7 (51.0-52.4)	51.3 (50.7-52.0)	.70
Male	48.3 (47.6-49.0)	48.3 (47.6-49.0)	48.7 (48.0-49.3)	
Self-reported race and ethnicity				
Asian	5.9 (5.4-6.4)	6.0 (5.4-6.5)	6.0 (5.5-6.6)	.69
Black	11.8 (10.9-12.6)	11.7 (10.8-12.6)	11.9 (11.0-12.8)	
Hispanic^b^	16.5 (15.2-17.8)	16.9 (15.6-18.2)	17.2 (15.9-18.6)	
White	63.2 (61.7-64.8)	62.8 (61.2-64.4)	62.1 (60.5-63.7)	
Other race^c^	2.6 (2.1-3.1)	2.7 (2.2-3.2)	2.8 (2.3-3.3)	
Income level				
Low (≤200% FPL)	30.1 (29.2-31.1)	27.5 (26.6-28.5)	27.8 (26.8-28.7)	<.001
Middle (201%-400% FPL)	30.9 (30.2-31.6)	29.5 (28.8-30.2)	29.1 (28.4-29.8)	
High (>400% FPL)	39.0 (38.0-40.0)	43.0 (42.0-44.0)	43.1 (42.0-44.2)	
Bachelor degree or higher	42.3 (41.3-43.2)	47.0 (46.0-48.1)	45.7 (44.8-46.7)	<.001
Employed	64.6 (63.8-65.3)	62.2 (61.5-63.0)	63.5 (62.8-64.3)	<.001
Clinical comorbidities				
Hypertension	31.7 (30.9-32.4)	31.5 (30.8-32.2)	32.0 (31.3-32.7)	.49
Hyperlipidemia	24.9 (24.2-25.5)	26.9 (26.3-27.6)	27.3 (26.6-27.9)	<.001
Diabetes	9.3 (9.0-9.7)	9.6 (9.2-10.0)	9.6 (9.2-10.0)	.54
Myocardial infarction	3.1 (2.9-3.4)	3.0 (2.8-3.3)	3.0 (2.7-3.2)	.50
Stroke	3.1 (2.9-3.3)	2.8 (2.6-3.0)	2.8 (2.6-3.1)	.14
COPD	4.6 (4.3-4.9)	4.6 (4.3-4.9)	4.6 (4.3-4.9)	.97
Cancer	9.5 (9.1-9.9)	9.8 (9.4-10.2)	9.6 (9.2-9.9)	.52
Rural	14.3 (13.1-15.6)	13.3 (12.1-14.5)	13.8 (12.6-15.0)	.23
US region^d^				
Northeast	17.8 (16.7-18.9)	17.5 (16.3-18.6)	17.6 (16.5-18.7)	.98
Midwest	21.0 (19.6-22.5)	20.8 (19.5-22.1)	20.7 (19.3-22.0)	
South	37.7 (35.9-39.5)	37.9 (36.3-39.6)	38.1 (36.3-39.9)	
West	23.5 (21.8-25.3)	23.8 (22.2-25.4)	23.6 (21.8-25.4)	

Abbreviations: COPD, chronic obstructive pulmonary disease; FPL, federal poverty level; NA, not applicable.

^a^ Nationally representative estimates are shown based on survey weights for the 2019, 2021, and 2022 National Health Interview Survey.

^b^ Includes all races that reported Hispanic ethnicity.

^c^ The other race category includes American Indian and Alaska Native, Native Hawaiian and Other Pacific Islander, multiracial, and other race.

^d^ US Census Bureau regions.

### Health Care Affordability

**Figure 1.**
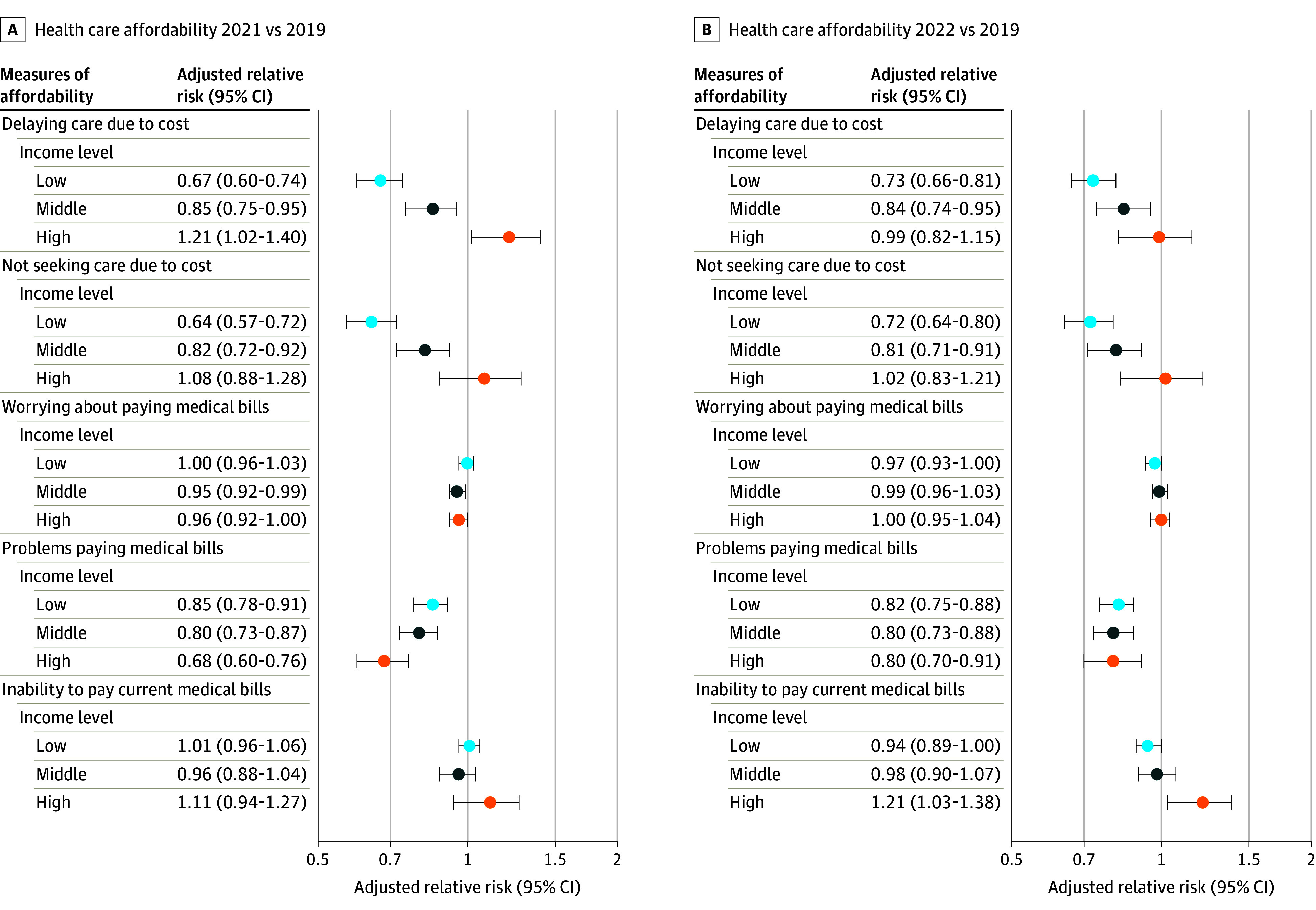
Forest Plot of Health Care Affordability by Income Level, 2021 and 2022 vs 2019 Measures of health care affordability in 2021 vs 2019 (A) and in 2022 vs 2019 (B) for low-income, middle-income, and high-income adults. Models adjusted for age and sex. Inability to pay current medical bills was reported among individuals who had problems paying medical bills within the last year. Error bars indicate 95% CIs.

Low-income adults reported worse health care affordability across all measures compared with the other income groups (Table 2). Compared with prepandemic levels, during the COVID-19 pandemic, low-income adults were less likely to delay medical care (2022: 11.2%; 95% CI, 10.3-12.1; 2019: 15.4%; 95% CI, 14.3-16.4; adjusted relative risk [aRR], 0.73; 95% CI, 0.66-0.81) or avoid care (2022: 10.7%; 95% CI, 9.7-11.6; 2019: 14.9%; 95% CI, 13.8-15.9; aRR, 0.72; 95% CI, 0.64-0.80) due to cost, while high-income adults experienced no change (Table 2; Figure 1). As a result, income-based disparities between these groups significantly narrowed over the course of the COVID-19 pandemic. In addition, low-income adults were less likely to experience problems paying medical bills (2022: 17.1%; 95% CI, 16.0-18.2; 2019: 21.0%; 95% CI, 19.8-22.2; aRR, 0.82; 95% CI, 0.75-0.88) but no change in worrying about medical bills. These patterns were similar in the high-income group. There was no change in the inability to pay medical bills among low-income adults, although high-income adults experienced greater difficulty paying medical bills during the COVID-19 pandemic (2022: 50.0%; 95% CI, 45.2-54.8; 2019: 41.3%; 95% CI, 36.9-45.8; aRR, 1.21; 95% CI, 1.03-1.38). Similar changes in outcomes were observed when comparing 2021 with 2019. In addition, changes in outcomes for middle-income adults were similar to that of low-income adults across both 2021 and 2022 (Table 2; Figure 1). Unadjusted risk differences for measures of health care affordability are shown in eTable 3 in Supplement 1.

**Table 2.** Health Care Affordability by Income Level, 2021 and 2022 vs 2019

Income level	% (95% CI)			2021 vs 2019		2022 vs 2019	
	2019 (Pre–COVID-19 pandemic)	2021 (During COVID-19 pandemic)	2022 (During COVID-19 pandemic)	aRR^a^	*P* for interaction^b^	aRR^a^	*P* for interaction^b^
Delaying care due to cost							
Low	15.4 (14.3-16.4)	10.2 (9.4-11.1)	11.2 (10.3-12.1)	0.67 (0.60-0.74)	<.001	0.73 (0.66-0.81)	<.001
Middle	9.8 (9.0-10.6)	8.3 (7.6-9.0)	8.2 (7.5-9.0)	0.85 (0.75-0.95)	<.001	0.84 (0.74-0.95)	.10
High	3.6 (3.2-4.0)	4.4 (3.9-4.8)	3.6 (3.2-4.0)	1.21 (1.02-1.40)	NA	0.99 (0.82-1.15)	NA
Not seeking care due to cost							
Low	14.9 (13.8-15.9)	9.5 (8.7-10.3)	10.7 (9.7-11.6)	0.64 (0.57-0.72)	<.001	0.72 (0.64-0.80)	<.001
Middle	8.7 (8.0-9.4)	7.1 (6.5-7.8)	7.0 (6.3-7.7)	0.82 (0.72-0.92)	.01	0.81 (0.71-0.91)	.04
High	2.9 (2.5-3.3)	3.1 (2.7-3.5)	3.0 (2.6-3.4)	1.08 (0.88-1.28)	NA	1.02 (0.83-1.21)	NA
Worrying about paying medical bills							
Low	56.4 (54.8-57.9)	56.1 (54.6-57.6)	54.4 (52.9-55.9)	1.00 (0.96-1.03)	.27	0.97 (0.93-1.00)	.24
Middle	52.0 (50.6-53.3)	49.5 (48.1-50.9)	51.5 (50.0-53.0)	0.95 (0.92-0.99)	.51	0.99 (0.96-1.03)	.92
High	35.8 (34.7-36.9)	34.2 (33.2-35.3)	35.6 (34.5-36.7)	0.96 (0.92-1.00)	NA	1.00 (0.95-1.04)	NA
Problems paying medical bills							
Low	21.0 (19.8-22.2)	17.8 (16.7-18.9)	17.1 (16.0-18.2)	0.85 (0.78-0.91)	.01	0.82 (0.75-0.88)	.81
Middle	16.1 (15.1-17.1)	12.8 (12.0-13.7)	12.9 (12.0-13.9)	0.80 (0.73-0.87)	.03	0.80 (0.73-0.88)	.77
High	6.3 (5.8-6.9)	4.3 (3.9-4.7)	5.1 (4.6-5.6)	0.68 (0.60-0.76)	NA	0.80 (0.70-0.91)	NA
Inability to pay current medical bills^c^							
Low	71.3 (68.7-73.8)	71.7 (68.8-74.6)	67.1 (63.8-70.4)	1.01 (0.96-1.06)	.40	0.94 (0.89-1.00)	<.001
Middle	60.0 (56.8-63.1)	57.3 (53.6-60.9)	58.8 (55.0-62.6)	0.96 (0.88-1.04)	.11	0.98 (0.90-1.07)	.03
High	41.3 (36.9-45.8)	45.2 (40.1-50.3)	50.0 (45.2-54.8)	1.11 (0.94-1.27)	NA	1.21 (1.03-1.38)	NA

Abbreviations: aRR, adjusted relative risk; NA, not applicable.

^a^ Survey-weighted logistic regression models adjusted for participant age and sex.

^b^ Models included an interaction term for income level (low, middle, or high) and period (2019 vs 2021 or 2022) to assess for differential changes in outcomes by income level during the COVID-19 pandemic. High-income adults were the reference group.

^c^ Among individuals who reported having problems paying or being unable to pay any medical bills within the last year.

### Prescription Medication Affordability

**Figure 2.**
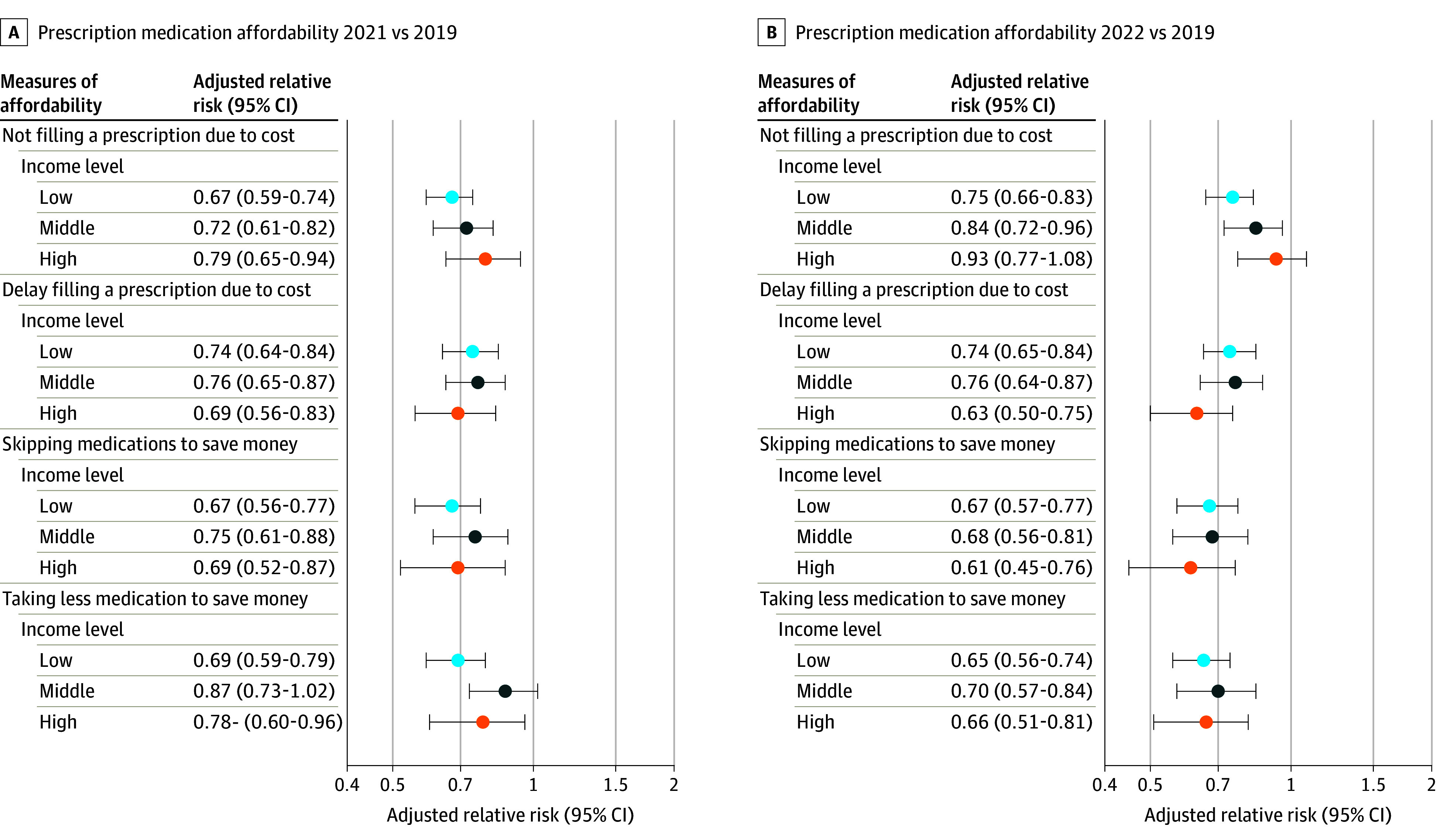
Forest Plot of Prescription Medication Affordability by Income Level, 2021 and 2022 vs 2019 Measures of prescription medication affordability in 2021 vs 2019 (A) and in 2022 vs 2019 (B) for low-income, middle-income, and high-income adults. Models adjusted for age and sex. Error bars indicate 95% CIs.

Low-income adults reported greater difficulties with prescription medication affordability throughout the study period compared with all other income groups (Table 3). During the COVID-19 pandemic, all 3 income groups experienced improved medication affordability across 4 self-reported measures (Table 3; Figure 2). Low-income adults had less problems filling prescriptions due to cost in 2022 (2022: 8.9%; 95% CI, 8.1-9.8; 2019: 12.0%; 95% CI, 11.1-12.9; aRR, 0.75; 95% CI, 0.66-0.83) compared with 2019, while high-income adults reported no change (2022: 2.7%; 95% CI, 2.3-3.0; 2019: 2.9%; 95% CI, 2.6-3.2; aRR, 0.93; 95% CI, 0.77-1.08) (*P* for interaction = .02). Additionally, low-income adults were less likely to delay filling prescriptions due to cost (2022: 9.4%; 95% CI, 8.4-10.4; 2019: 12.7%; 95% CI, 11.6-13.9; aRR, 0.74; 95% CI, 0.65-0.84), skip medications to save money (2022: 6.7%; 95% CI, 5.9-7.6; 2019: 10.1%; 95% CI, 9.1-11.1; aRR, 0.67; 95% CI, 0.57-0.77), or take less medications to save money (2022: 7.3%; 95% CI, 6.4-8.1; 2019: 11.2%; 95% CI, 10.1-12.2; aRR, 0.65; 95% CI, 0.56-0.74). Changes in these measures were similar for middle-income and high-income adults in 2022 and across all 3 income groups in 2021. Unadjusted risk differences for measures of prescription medication affordability are shown in eTable 4 in Supplement 1.

**Table 3.** Prescription Medication Affordability by Income Level, 2021 and 2022 vs 2019

Income level	% (95% CI)			2021 vs 2019		2022 vs 2019	
	2019 (Pre–COVID-19 pandemic)	2021 (During COVID-19 pandemic)	2022 (During COVID-19 pandemic)	aRR^a^	*P* for interaction^b^	aRR^a^	*P* for interaction^b^
Not filling a prescription due to cost							
Low	12.0 (11.1-12.9)	8.1 (7.3-8.8)	8.9 (8.1-9.8)	0.67 (0.59-0.74)	.06	0.75 (0.66-0.83)	.02
Middle	7.2 (6.6-7.9)	5.2 (4.6-5.8)	6.1 (5.4-6.8)	0.72 (0.61-0.82)	.33	0.84 (0.72-0.96)	.31
High	2.9 (2.6-3.2)	2.3 (2.0-2.6)	2.7 (2.3-3.0)	0.79 (0.65-0.94)	NA	0.93 (0.77-1.08)	NA
Delay filling a prescription due to cost^c^							
Low	12.7 (11.6-13.9)	9.4 (8.4-10.4)	9.4 (8.4-10.4)	0.74 (0.64-0.84)	.72	0.74 (0.65-0.84)	.27
Middle	8.8 (8.0-9.7)	6.6 (5.9-7.4)	6.7 (5.9-7.4)	0.76 (0.65-0.87)	.52	0.76 (0.64-0.87)	.18
High	3.8 (3.3-4.3)	2.6 (2.2-3.0)	2.4 (2.0-2.8)	0.69 (0.56-0.83)	NA	0.63 (0.50-0.75)	NA
Skipping medications to save money^c^							
Low	10.1 (9.1-11.1)	6.7 (5.9-7.5)	6.7 (5.9-7.6)	0.67 (0.56-0.77)	.66	0.67 (0.57-0.77)	.67
Middle	6.5 (5.8-7.2)	4.8 (4.1-5.5)	4.4 (3.8-5.1)	0.75 (0.61-0.88)	.68	0.68 (0.56-0.81)	.51
High	2.4 (2.0-2.8)	1.7 (1.3-2.0)	1.5 (1.2-1.8)	0.69 (0.52-0.87)	NA	0.61 (0.45-0.76)	NA
Taking less medication to save money^c^							
Low	11.2 (10.1-12.2)	7.6 (6.8-8.5)	7.3 (6.4-8.1)	0.69 (0.59-0.79)	.27	0.65 (0.56-0.74)	.71
Middle	6.8 (6.0-7.6)	5.9 (5.2-6.6)	4.8 (4.1-5.5)	0.87 (0.73-1.02)	.45	0.70 (0.57-0.84)	.75
High	2.7 (2.3-3.1)	2.1 (1.7-2.5)	1.8 (1.5-2.1)	0.78 (0.60-0.96)	NA	0.66 (0.51-0.81)	NA

Abbreviations: aRR, adjusted relative risk; NA, not applicable.

^a^ Survey-weighted logistic regression models adjusted for participant age and sex.

^b^ Models included an interaction term for income level (low, middle, or high) and period (2019 vs 2021 or 2022) to assess for differential changes in outcomes by income level during the COVID-19 pandemic. High-income adults were the reference group.

^c^ Among individuals taking prescription medications.

### Additional Analysis

Health insurance coverage increased only among low-income adults (2022: 81.8%; 95% CI, 80.4-83.3; 2019: 78.4%; 95% CI, 77.0-79.8; aRR, 1.04; 95% CI, 1.02-1.06) during the COVID-19 pandemic due to an increase in public health insurance, while health care use decreased modestly across all income groups (eTables 5 to 7 in Supplement 1). To understand whether changes in insurance coverage and health care use contributed to observed changes in health care and prescription medication affordability, we repeated our main analysis after also including these variables in our models. Changes in measures of health care and prescription medication affordability across income groups were largely similar after additionally adjusting for health insurance coverage and health care use (eTables 8 and 9 in Supplement 1). In addition, changes across all outcomes were similar to our primary analysis after restricting the study population to working-age adults (age 18 to 64 years) (eTables 10 and 11 in Supplement 1). Findings for the subgroup of adults 65 years and older and all US adults 18 years and older are shown in eTables 12 to 15 in Supplement 1.

## Discussion

In this US national study, we found that health care affordability improved for low-income adults during the COVID-19 pandemic in 2021 and 2022 compared with prepandemic levels in 2019 and largely remained unchanged for those with high incomes. As a result, income-based inequities narrowed across many measures of health care affordability. In addition, prescription medication affordability improved for all income groups during the COVID-19 pandemic. These changes in health care and prescription medication affordability during the COVID-19 pandemic were not explained by health insurance coverage or health care use.

Despite initial increases in unemployment and economic loss, we found that medical care and prescription medications became more affordable for US adults during the COVID-19 pandemic. Our findings extend upon prior studies focused on the initial months of the COVID-19 pandemic in 2020 by demonstrating that improvements in health care and medication affordability persisted in later years of the pandemic (2021 and 2022).^19,20,21^ Notably, this was a change from the prepandemic trend of health care affordability, which had been stable if not worsening.^17,22^ The results are also a stark contrast to prior historic economic downturns, like the Great Recession, which saw a significant decline in health care affordability that persisted for several years.^23,24^

A major finding of this study is that low-income adults in the US reported as much or greater improvement in health care affordability compared with their high-income counterparts. To our knowledge, this is one of the first studies to evaluate changes in health care and medication affordability among low-income adults in 2021 and 2022, a period when many policies that bolstered the safety net were enacted or expanded. Historically, low income has been associated with a greater likelihood of being uninsured and trouble paying medical bills, leading to higher rates of delayed or foregone medical care.^6,7,9,17,19,25^ Income level and insurance status are also strong predictors of cost-related medication nonadherence.^26^ Although the economic downfall from the COVID-19 pandemic disproportionately impacted lower-paid workers and threatened to widen income-based disparities in health care affordability,^2,3,27^ we observed the opposite pattern in our study—income-based inequities across several measures of health care affordability narrowed.

One potential explanation for the improvement in health care affordability among low-income adults is the direct economic aid granted through federal safety-net policies. The US government provided economic impact funds that offset income losses for 80% of displaced workers and increased funding to policies supporting low-income adults, such as the Supplemental Nutrition Assistance Program, which doubled benefits between 2019 and 2022.^12,28^ Millions of adults in the US also received thousands of dollars through stimulus checks and child tax credits.^29^ These efforts resulted in posttax incomes rising in 2020 compared with 2019, particularly among low-income workers.^30^

Our study also explored several other potential explanations for improvements in health care and medication affordability. We found that public health insurance coverage (eg, Medicaid) during the COVID-19 pandemic remained high and actually improved for low-income adults, likely due to federal policies that increased Medicaid funding in exchange for states suspending eligibility redeterminations and maintaining coverage for enrollees through the end of the public health emergency.^31,32,33^ The federal government also temporarily expanded eligibility for tax credits and created an open enrollment period for Affordable Care Act marketplace health insurance plans.^11^ While growth in health insurance coverage during the COVID-19 pandemic did not explain the improvements in health care and prescription medication affordability observed in our study, these federal policies likely prevented coverage and affordability from worsening for low-income adults. Our findings suggest that the improvements in affordability were not due to reductions in acute care use. The early phase of the COVID-19 pandemic saw a dramatic decrease in emergency department visits, hospitalizations, and ambulatory visits with a shift toward telemedicine.^34,35,36,37^ This shift in care was relatively transient, however, with health care visits increasing by late 2020.^35,38^ We found that acute care use remained modestly lower across all income groups as the COVID-19 pandemic went on in 2021 and 2022. Other considerations for the observed improvements in health care and prescription medication affordability, which our study was not able to account for, include US economic growth during the second half of the COVID-19 pandemic and/or changes in perceptions of the importance of medical care.

This study has important health policy implications as the US emerges from the COVID-19 pandemic. May 11, 2023, marked the end of the COVID-19 pandemic public health emergency, and many of the federal policies that may have contributed to the improvements in health care affordability that we observed during the pandemic, particularly among low-income adults, are coming to an end. Economic impact funds have been discontinued, and the reinstatement of Medicaid eligibility requirements in 2023 has already led to the disenrollment of millions of beneficiaries.^39,40^ This unwinding of federal support threatens to reverse the improvements in affordability of care and the narrowing of income-based disparities in 2021 and 2022, which could have major implications on the health of low-income adults as the US emerges from the COVID-19 pandemic.

### Limitations

This study has limitations. First, measures of health care and medication affordability with NHIS are self-reported and measure perceptions of affordability rather than true costs or spending. Second, the pre-post study design makes it difficult to interpret if the changes in affordability were due to intervening factors or a continuation of a larger trend. However, prior studies showed that health care affordability had been stable if not worsening prior to the COVID-19 pandemic.^17,22^ Third, pandemic-related disruptions in NHIS data acquisition resulted in a greater reliance on telephone rather than face-to-face interviews in 2021 and may have introduced mode of administration bias. However, our findings were consistent for 2022—a year in which data acquisition was more similar to 2019. Fourth, we were unable to capture changes in health care and prescription medication affordability after the unwinding of federal policies as many came to an end in 2023; this remains an important area for future research.

## Conclusions

In this US national study, we found that health care affordability improved for low-income adults during the COVID-19 pandemic in 2021 and 2022 compared with 2019 and largely remained unchanged for those with high incomes. As a result, income-based disparities narrowed across multiple measures of health care affordability. In addition, prescription medication affordability improved for both low-income and higher-income adults. Our findings have critically important public health implications as many COVID-19 pandemic–related state and federal safety-net policies come to an end.

## Notes

Supplement 1. **eTable 1.** Unweighted Baseline Characteristics of US Adults by Year **eTable 2.** Baseline Characteristics of US Adults by Income Level in 2019 (Prepandemic) and 2021/2022 (Pandemic) **eTable 3.** Health Care Affordability by Income Level (Unadjusted Risk Differences), 2021/2022 vs 2019 **eTable 4.** Prescription Medication Affordability by Income Level (Unadjusted Risk Differences), 2021/2022 vs 2019 **eTable 5.** Health Insurance Coverage by Income Level, 2021/2022 vs 2019 **eTable 6.** Health Insurance Type by Income Level, 2021/2022 vs 2019 **eTable 7.** Health Care Use by Income Level, 2021/2022 vs 2019 **eTable 8.** Health Care and Prescription Medication Affordability by Income Level, 2021 vs 2019 Adjusting for Health Insurance and Health Care Use **eTable 9.** Health Care and Prescription Medication Affordability by Income Level, 2022 vs 2019 Adjusting for Health Insurance and Health Care Use **eTable 10.** Health Care Affordability by Income Level Among Adults Aged 18-64 Years, 2021/2022 vs 2019 **eTable 11.** Prescription Medication Affordability by Income Level Among Adults Aged 18-64 Years, 2021/2022 vs 2019 **eTable 12.** Health Care Affordability by Income Level Among Adults Aged ≥65 Years, 2021/2022 vs 2019 **eTable 13.** Prescription Medication Affordability by Income Level Among Adults Aged ≥65 Years, 2021/2022 vs 2019 **eTable 14.** Health Care Affordability Among US Adults, 2021/2022 vs 2019 **eTable 15.** Prescription Medication Affordability Among US Adults, 2021/2022 vs 2019

Supplement 2. Data Sharing Statement
