# Supplementary material for: Changes in Health Care and Prescription Medication Affordability in the US During the COVID-19 Pandemic
Source: JAMA Health Forum. 2024 Jun 30;5(6.9):e241939. doi: 10.1001/jamahealthforum.2024.1939 (PMC11215556; doi:10.1001/jamahealthforum.2024.1939)
Supplement: Supplement 1. — eTable 1. Unweighted Baseline Characteristics of US Adults by Year eTable 2. Baseline Characteristics of US Adults by Income Level in 2019 (Prepandemic) and 2021/2022 (Pandemic) eTable 3. Health Care Affordability by Income Level (Unadjusted Risk Differences), 2021/2022 vs 2019 eTable 4. Prescription Medication Affordability by Income Level (Unadjusted Risk Differences), 2021/2022 vs 2019 eTable 5. Health Insurance Coverage by Income Level, 2021/2022 vs 2019 eTable 6. Health Insurance Type by Income Level, 2021/2022 vs 2019 eTable 7. Health Care Use by Income Level, 2021/2022 vs 2019 eTable 8. Health Care and Prescription Medication Affordability by Income Level, 2021 vs 2019 Adjusting for Health Insurance and Health Care Use eTable 9. Health Care and Prescription Medication Affordability by Income Level, 2022 vs 2019 Adjusting for Health Insurance and Health Care Use eTable 10. Health Care Affordability by Income Level Among Adults Aged 18-64 Years, 2021/2022 vs 2019 eTable 11. Prescription Medication Affordability by Income Level Among Adults Aged 18-64 Years, 2021/2022 vs 2019 eTable 12. Health Care Affordability by Income Level Among Adults Aged ≥65 Years, 2021/2022 vs 2019 eTable 13. Prescription Medication Affordability by Income Level Among Adults Aged ≥65 Years, 2021/2022 vs 2019 eTable 14. Health Care Affordability Among US Adults, 2021/2022 vs 2019 eTable 15. Prescription Medication Affordability Among US Adults, 2021/2022 vs 2019 [file jamahealthforum-e241939-s001.pdf]

Supplemental Online Content

Mein SA, Marinacci LX, Zheng ZN, Ahmad I, Wadhera RK. Changes in health care and prescription medication affordability in the US during the COVID-19 pandemic. *JAMA Health Forum.* 2024;5(6.9):e241939. doi:10.1001/jamahealthforum.2024.1939

eTable 1. Unweighted Baseline Characteristics of US Adults by Year
eTable 2. Baseline Characteristics of US Adults by Income Level in 2019 (Prepandemic) and 2021/2022 (Pandemic)
eTable 3. Health Care Affordability by Income Level (Unadjusted Risk Differences), 2021/2022 vs 2019
eTable 4. Prescription Medication Affordability by Income Level (Unadjusted Risk Differences), 2021/2022 vs 2019
eTable 5. Health Insurance Coverage by Income Level, 2021/2022 vs 2019
eTable 6. Health Insurance Type by Income Level, 2021/2022 vs 2019
eTable 7. Health Care Use by Income Level, 2021/2022 vs 2019
eTable 8. Health Care and Prescription Medication Affordability by Income Level, 2021 vs 2019 Adjusting for Health Insurance and Health Care Use
eTable 9. Health Care and Prescription Medication Affordability by Income Level, 2022 vs 2019 Adjusting for Health Insurance and Health Care Use
eTable 10. Health Care Affordability by Income Level Among Adults Aged 18-64 Years, 2021/2022 vs 2019
eTable 11. Prescription Medication Affordability by Income Level Among Adults Aged 18-64 Years, 2021/2022 vs 2019
eTable 12. Health Care Affordability by Income Level Among Adults Aged ≥65 Years, 2021/2022 vs 2019
eTable 13. Prescription Medication Affordability by Income Level Among Adults Aged ≥65 Years, 2021/2022 vs 2019
eTable 14. Health Care Affordability Among US Adults, 2021/2022 vs 2019
eTable 15. Prescription Medication Affordability Among US Adults, 2021/2022 vs 2019

This supplementary material has been provided by the authors to give readers additional information about their work.

© 2024 Mein SA et al. *JAMA Health Forum*.

eTable 1. Unweighted Baseline Characteristics of US Adults by Year

	US population, % (95% CI)			
	**2019 (Pre-pandemic)**	**2021 (Pandemic)**	**2022 (Pandemic)**	***P* value**
Unweighted population size, n	31,997	29,482	27,651	-
Age, mean (SD)	52.0 (0.10)	52.5 (0.11)	52.9 (0.11)	0.04
Female	54.0 (53.4, 54.5)	54.6 (54.1, 55.2)	54.4 (53.8, 55.0)	0.70
Race and ethnicity				
Non-Hispanic White	68.5 (68.0, 69.0)	66.7 (66.1, 67.2)	66.0 (65.4, 66.5)	0.69
Non-Hispanic Black	10.9 (10.5, 11.2)	10.7 (10.4, 11.1)	11.3 (10.9, 11.6)	
Non-Hispanic Asian	5.2 (4.9, 5.4)	6.1 (5.9, 6.4)	6.0 (5.7, 6.3)	
Hispanic	13.0 (12.6, 13.3)	13.8 (13.4, 14.2)	14.3 (13.8, 14.7)	
Other	2.5 (2.3, 2.7)	2.6 (2.4, 2.8)	2.5 (2.3, 2.7)	
Income level				
Low (≤200% FPL)	29.3 (28.8, 29.8)	27.3 (26.8, 27.8)	27.8 (27.3, 28.3)	<0.001
Middle (201-400% FPL)	30.0 (29.5, 30.5)	28.9 (28.4, 29.4)	28.9 (28.3, 29.4)	
High (>400% FPL)	40.7 (40.2, 41.2)	43.8 (43.2, 44.3)	43.3 (42.7, 43.9)	
Bachelor’s degree or higher	48.5 (48.0, 49.1)	51.5 (50.9, 52.0)	51.0 (50.5, 51.6)	<0.001
Employment	60.2 (59.7, 60.8)	57.7 (57.2, 58.3)	58.0 (57.4, 58.6)	<0.001
Clinical comorbidities				
Hypertension	35.9 (35.4, 36.5)	36.2 (35.7, 36.8)	36.8 (36.2, 37.3)	0.49
Hyperlipidemia	28.8 (28.3, 29.3)	30.9 (30.4, 31.4)	31.7 (31.1, 32.2)	<0.001
Diabetes mellitus	10.5 (10.2, 10.8)	10.6 (10.3, 11.0)	10.7 (10.3, 11.0)	0.54
Myocardial infarction	3.9 (3.7, 4.1)	3.7 (3.4, 3.9)	3.7 (3.5, 3.9)	0.50
Stroke	3.8 (3.6, 4.0)	3.4 (3.2, 3.6)	3.7 (3.4, 3.9)	0.14
COPD	5.6 (5.3, 5.8)	5.7 (5.5, 6.0)	5.5 (5.2, 5.8)	0.97
Cancer	12.0 (11.7, 12.4)	12.4 (12.0, 12.8)	12.4 (12.0, 12.8)	0.52
Rural	15.9 (15.5, 16.3)	14.5 (14.1, 14.9)	15.6 (15.2, 16.0)	0.23
US region[a]				
Northeast	16.9 (16.5, 17.3)	16.2 (15.8, 16.6)	16.6 (16.2, 17.0)	0.98
Midwest	22.2 (21.7, 22.7)	21.5 (21.0, 21.9)	21.8 (21.3, 22.3)	
South	36.5 (36.0, 37.0)	36.4 (35.8, 36.9)	36.8 (36.2, 37.4)	
West	24.4 (23.9, 24.9)	25.9 (25.4, 26.4)	24.8 (24.3, 25.3)	

[a] US Census Bureau regions.

© 2024 Mein SA et al. *JAMA Health Forum*.

eTable 2. Baseline Characteristics of US Adults by Income Level in 2019 (Prepandemic) and 2021/2022 (Pandemic)

Characteristics	US population, % (95% CI)								
	Low-income[a] (n=25,127)			Middle-income[b] (n=26,092)			High-income[c] (n=37,911)		
	**2019**	**2021**	**2022**	**2019**	**2021**	**2022**	**2019**	**2021**	**2022**
Weighted population size[d], n	75,558,277	69,650,362	70,872,289	77,524,847	74,663,721	74,401,978	97,816,026	108,827,265	110,070,436
Age group, mean (SD)	46.3 (0.29)	47.0 (0.32)	47.0 (0.30)	47.5 (0.25)	48.1 (0.26)	48.3 (0.26)	49.0 (0.21)	48.9 (0.21)	48.7(0.21)
Female	56.1 (54.7, 57.5)	57.9 (56.6, 59.2)	56.4 (55.1, 57.8)	51.1 (49.9, 52.3)	52.0 (50.7, 53.3)	51.6 (50.3, 52.9)	48.8 (47.8, 49.8)	47.5 (46.5, 48.5)	47.9 (46.9, 48.9)
Race and ethnicity									
Non-Hispanic White	47.2 (45.0, 49.5)	45.5 (43.1, 47.9)	45.8 (43.5, 48.1)	62.6 (60.6, 64.5)	61.2 (59.3, 63.2)	60.8 (58.8, 62.7)	76.1 (74.7, 77.5)	74.9 (73.5, 76.3)	73.4 (72.0, 74.9)
Non-Hispanic Black	17.3 (15.7, 19.0)	17.9 (16.2, 19.7)	17.9 (16.1, 19.6)	12.2 (11.1, 13.4)	12.3 (11.1, 13.5)	12.1 (10.9, 13.3)	7.0 (6.3, 7.8)	7.3 (6.4, 8.1)	7.9 (7.1, 8.7)
Non-Hispanic Asian	5.4 (4.7, 6.2)	5.1 (4.3, 5.9)	5.4 (4.6, 6.1)	5.2 (4.5, 6.0)	5.4 (4.7, 6.1)	5.4 (4.7, 6.1)	6.8 (6.0, 7.6)	6.9 (6.2, 7.6)	6.9 (6.1, 7.7)
Hispanic	26.7 (24.4, 28.9)	27.8 (25.4, 30.2)	27.6 (25.3, 29.8)	17.2 (15.6, 18.8)	18.4 (16.8, 20.0)	18.7 (17.0, 20.4)	8.2 (7.4, 9.1)	9.0 (8.1, 9.9)	9.6 (8.6, 10.5)
Other	3.4 (2.4, 4.3)	3.6 (2.1, 5.1)	3.5 (2.4, 4.6)	2.8 (2.1, 3.4)	2.7 (2.3, 3.2)	3.0 (2.4, 3.6)	1.9 (1.6, 2.2)	2.0 (1.7, 2.4)	2.2 (1.9, 2.5)
Bachelor's degree or higher	20.4 (19.4, 21.5)	21.6 (20.4, 22.8)	20.7 (19.6, 21.8)	37.6 (36.4, 38.9)	40.8 (39.5, 42.2)	38.7 (37.4, 40.1)	62.7 (61.5, 63.9)	67.5 (66.3, 68.6)	66.5 (65.4, 67.6)
Employment	49.6 (48.2, 51.0)	46.5 (45.0, 47.9)	46.3 (44.8, 47.8)	66.0 (64.7, 67.2)	61.3 (60.0, 62.6)	63.4 (62.2, 64.7)	75.0 (74.1, 75.8)	72.7 (71.7, 73.8)	74.5 (73.6, 75.4)
Clinical Comorbidities									
Hypertension	35.1 (33.8, 36.3)	34.6 (33.3, 35.9)	36.0 (34.7, 37.4)	32.5 (31.4, 33.7)	32.7 (31.5, 33.9)	32.7 (31.5, 33.9)	28.3 (27.3, 29.3)	28.6 (27.6, 29.6)	29.0 (28.0, 29.9)
Hyperlipidemia	25.2 (24.1, 26.4)	26.5 (25.3, 27.7)	27.5 (26.4, 28.7)	24.1 (23.1, 25.2)	26.8 (25.7, 27.9)	27.1 (25.9, 28.3)	25.1 (24.3, 26.0)	27.3 (26.4, 28.2)	27.2 (26.3, 28.2)
Diabetes mellitus	12.5 (11.7, 13.3)	12.9 (12.1, 13.8)	12.7 (11.9, 13.5)	9.9 (9.2, 10.6)	10.7 (9.9, 11.4)	10.4 (9.6, 11.2)	6.4 (6.0, 6.9)	6.8 (6.3, 7.3)	7.0 (6.4, 7.5)

© 2024 Mein SA et al. *JAMA Health Forum*.

	US population, % (95% CI)								
	Low-income[*] (n=25,127)			Middle-income[†] (n=26,092)			High-income[‡] (n=37,911)		
	2019	2021	2022	2019	2021	2022	2019	2021	2022
Myocardial infarction	4.4 (3.9, 4.9)	4.4 (3.9, 4.9)	4.1 (3.6, 4.6)	3.3 (2.9, 3.6)	3.2 (2.8, 3.6)	3.1 (2.7, 3.5)	2.1 (1.8, 2.3)	2.1 (1.8, 2.4)	2.1 (1.9, 2.4)
Stroke	5.1 (4.5, 5.6)	4.6 (4.0, 5.1)	4.8 (4.2, 5.3)	3.0 (2.6, 3.3)	3.1 (2.7, 3.5)	3.0 (2.6, 3.4)	1.7 (1.4, 1.9)	1.5 (1.3, 1.7)	1.5 (1.3, 1.7)
COPD	7.5 (6.9, 8.1)	7.4 (6.7, 8.0)	8.1 (7.4, 8.9)	4.6 (4.1, 5.1)	5.1 (4.6, 5.6)	4.3 (3.8, 4.7)	2.4 (2.1, 2.7)	2.5 (2.2, 2.8)	2.5 (2.2, 2.8)
Cancer	8.5 (7.7, 9.2)	8.1 (7.4, 8.8)	8.3 (7.6, 9.0)	9.2 (8.5, 9.9)	9.7 (9.0, 10.4)	9.5 (8.8, 10.1)	10.5 (9.9, 11.1)	10.9 (10.3, 11.5)	10.4 (9.9, 11.0)
Rural	18.3 (16.4, 20.2)	17.2 (15.3, 19.1)	18.4 (16.4, 20.4)	15.8 (14.3, 17.3)	15.4 (13.8, 17.0)	16.3 (14.6, 17.9)	10.1 (8.9, 11.4)	9.4 (8.2, 10.7)	9.2 (8.1, 10.4)
US Region[e]									
Northeast	15.3 (13.9, 16.7)	15.2 (13.6, 16.7)	15.4 (14.0, 16.7)	16.5 (15.2, 17.8)	15.8 (14.3, 17.3)	15.7 (14.3, 17.0)	20.7 (19.2, 22.2)	20.1 (18.6, 21.6)	20.3 (18.7, 21.9)
Midwest	18.7 (17.1, 20.4)	18.8 (17.3, 20.3)	19.6 (17.9, 21.4)	22.0 (20.3, 23.7)	22.4 (20.8, 24.1)	21.7 (20.0, 23.4)	22.0 (20.2, 23.8)	20.9 (19.4, 22.5)	20.7 (19.0, 22.3)
South	42.9 (40.6, 45.2)	43.3 (40.8, 45.7)	42.9 (40.4, 45.3)	38.3 (36.2, 40.4)	37.9 (35.8, 40.0)	38.5 (36.3, 40.7)	33.1 (31.0, 35.3)	34.6 (32.6, 36.5)	34.8 (32.6, 37.0)
West	23.1 (20.8, 25.4)	22.8 (20.4, 25.2)	22.1 (19.6, 24.6)	23.2 (21.2, 25.2)	23.9 (22.0, 25.7)	24.1 (22.0, 26.3)	24.1 (22.1, 26.2)	24.4 (22.5, 26.3)	24.3 (22.1, 26.4)

Nationally representative estimates (%) are shown based on survey weights for the 2019, 2021, and 2022 NHIS.
[a] Low-income was defined as family income ≤ 200% federal poverty level.
[b] Middle-income was defined as family income >200% and ≤ 400% federal poverty level.
[c] High-income was defined as family income >400% federal poverty level.
[d] National estimates based on survey weights for the 2019, 2021 and 2022 NHIS.
[e] US Census Bureau regions.

© 2024 Mein SA et al. *JAMA Health Forum*.

eTable 3. Health Care Affordability by Income Level (Unadjusted Risk Differences), 2021/2022 vs 2019

	Income level	2019 (Pre-pandemic) % (95% CI)	2021 (Pandemic) % (95% CI)	Unadjusted risk difference (2021 vs 2019)	2022 (Pandemic) % (95% CI)	Unadjusted risk difference (2022 vs 2019)
Delaying care due to cost	Low	15.4 (14.3, 16.4)	10.2 (9.4, 11.1)	-5.1 (-6.5, -3.8)	11.2 (10.3, 12.1)	-4.2 (-5.5, -2.8)
	Middle	9.8 (9.0, 10.6)	8.3 (7.6, 9.0)	-1.5 (-2.5, -0.5)	8.2 (7.5, 9.0)	-1.6 (-2.7, -0.5)
	High	3.6 (3.2, 4.0)	4.4 (3.9, 4.8)	0.8 (0.1, 1.4)	3.6 (3.2, 4.0)	0.0 (-0.6, 0.5)
Not seeking care due to cost	Low	14.9 (13.8, 15.9)	9.5 (8.7, 10.3)	-5.4 (-6.7, -4.0)	10.7 (9.7, 11.6)	-4.2 (-5.6, -2.8)
	Middle	8.7 (8.0, 9.4)	7.1 (6.5, 7.8)	-1.6 (-2.5, -0.6)	7.0 (6.3, 7.7)	-1.7 (-2.7, -0.7)
	High	2.9 (2.5, 3.3)	3.1 (2.7, 3.5)	0.2 (-0.3, 0.8)	3.0 (2.6, 3.4)	0.1 (-0.5, 0.6)
Worry about paying medical bills	Low	56.4 (54.8, 57.9)	56.1 (54.6, 57.6)	-0.3 (-2.3, 1.8)	54.4 (52.9, 55.9)	-1.9 (-4.0, 0.1)
	Middle	52.0 (50.6, 53.3)	49.5 (48.1, 50.9)	-2.5 (-4.3, -0.7)	51.5 (50.0, 53.0)	-0.5 (-2.3, 1.4)
	High	35.8 (34.7, 36.9)	34.2 (33.2, 35.3)	-1.5 (-3.0, 0.0)	35.6 (34.5, 36.7)	-0.2 (-1.7, 1.4)
Problems paying medical bills	Low	21.0 (19.8, 22.2)	17.8 (16.7, 18.9)	-3.2 (-4.7, -1.7)	17.1 (16.0, 18.2)	-3.9 (-5.4, -2.4)
	Middle	16.1 (15.1, 17.1)	12.8 (12.0, 13.7)	-3.2 (-4.5, -2.0)	12.9 (12.0, 13.9)	-3.1 (-4.5, -1.8)
	High	6.3 (5.8, 6.9)	4.3 (3.9, 4.7)	-2.1 (-2.7, -1.4)	5.1 (4.6, 5.6)	-1.2 (-2.0, -0.5)
Inability to pay current medical bills[a]	Low	71.3 (68.7, 73.8)	71.7 (68.8, 74.6)	0.5 (-3.3, 4.3)	67.1 (63.8, 70.4)	-4.1 (-8.2, -0.1)
	Middle	60.0 (56.8, 63.1)	57.3 (53.6, 60.9)	-2.7 (-7.4, 2.0)	58.8 (55.0, 62.6)	-1.2 (-6.3, 4.0)
	High	41.3 (36.9, 45.8)	45.2 (40.1, 50.3)	3.9 (-2.7, 10.5)	50.0 (45.2, 54.8)	8.7 (2.0, 15.3)

[a] Among individuals who reported having problems paying or being unable to pay any medical bills within the last year.

© 2024 Mein SA et al. *JAMA Health Forum*.

eTable 4. Prescription Medication Affordability by Income Level (Unadjusted Risk Differences), 2021/2022 vs 2019

	**Income level**	**2019 (Pre-pandemic) % (95% CI)**	**2021 (Pandemic) % (95% CI)**	**Unadjusted relative risk (2021 vs 2019)**	**2022 (Pandemic) % (95% CI)**	**Unadjusted relative risk (2022 vs 2019)**
Not filling prescriptions due to cost	Low	12.0 (11.1, 12.9)	8.1 (7.3, 8.8)	-3.9 (-5.1, -2.8)	8.9 (8.1, 9.8)	-3.0 (-4.2, -1.9)
	Middle	7.2 (6.6, 7.9)	5.2 (4.6, 5.8)	-2.0 (-2.9, -1.2)	6.1 (5.4, 6.8)	-1.2 (-2.1, -0.2)
	High	2.9 (2.6, 3.2)	2.3 (2.0, 2.6)	-0.6 (-1.1, -0.2)	2.7 (2.3, 3.0)	-0.2 (-0.7, 0.2)
Delays in filling prescriptions due to cost[a]	Low	12.7 (11.6, 13.9)	9.4 (8.4, 10.4)	-3.4 (-4.8, -1.9)	9.4 (8.4, 10.4)	-3.3 (-4.7, -1.9)
	Middle	8.8 (8.0, 9.7)	6.6 (5.9, 7.4)	-2.2 (-3.3, -1.1)	6.7 (5.9, 7.4)	-2.2 (-3.3, -1.0)
	High	3.8 (3.3, 4.3)	2.6 (2.2, 3.0)	-1.2 (-1.8, -0.6)	2.4 (2.0, 2.8)	-1.4 (-2.1, -0.8)
Skipping doses of medications to save money[a]	Low	10.1 (9.1, 11.1)	6.7 (5.9, 7.5)	-3.4 (-4.7, -2.1)	6.7 (5.9, 7.6)	-3.3 (-4.6, -2.1)
	Middle	6.5 (5.8, 7.2)	4.8 (4.1, 5.5)	-1.7 (-2.7, -0.7)	4.4 (3.8, 5.1)	-2.1 (-3.1, -1.1)
	High	2.4 (2.0, 2.8)	1.7 (1.3, 2.0)	-0.8 (-1.3, -0.3)	1.5 (1.2, 1.8)	-1.0 (-1.5, -0.5)
Taking less medication to save money[a]	Low	11.2 (10.1, 12.2)	7.6 (6.8, 8.5)	-3.5 (-4.8, -2.2)	7.3 (6.4, 8.1)	-3.9 (-5.2, -2.6)
	Middle	6.8 (6.0, 7.6)	5.9 (5.2, 6.6)	-0.9 (-2.0, 0.1)	4.8 (4.1, 5.5)	-2.0 (-3.1, -1.0)
	High	2.7 (2.3, 3.1)	2.1 (1.7, 2.5)	-0.6 (-1.2, -0.1)	1.8 (1.5, 2.1)	-0.9 (-1.4, -0.4)

[a] Among individuals taking prescription medications.

© 2024 Mein SA et al. *JAMA Health Forum*.

eTable 5. Health Insurance Coverage by Income Level, 2021/2022 vs 2019

Income Level	2019 (Pre-pandemic) % (95% CI)	2021 (Pandemic) % (95% CI)	Adjusted relative risk (2021 vs 2019)[a]	*P* value of interaction term[b]	2022 (Pandemic) % (95% CI)	Adjusted relative risk (2022 vs 2019)[a]	*P* value of interaction term[b]
Low	78.4 (77.0, 79.8)	81.0 (79.7, 82.3)	1.03 (1.01, 1.05)	0.27	81.8 (80.4, 83.3)	1.04 (1.02, 1.06)	0.12
Middle	87.9 (87.0, 88.8)	89.2 (88.3, 90.1)	1.01 (1.00, 1.03)	0.43	88.9 (88.0, 89.8)	1.01 (0.99, 1.02)	0.80
High	96.3 (95.9, 96.7)	96.4 (96.0, 96.8)	1.00 (0.99, 1.01)	-	96.5 (96.0, 96.9)	1.00 (1.00, 1.01)	-

[a] Survey-weighted logistic regression models adjusted for participant age and sex.
[b] Models included an interaction term for income-level (low, middle, high) and period (2019 versus 2021/2022), to assess for differential changes in outcomes by income-level during the pandemic. High-income adults were the reference group.

© 2024 Mein SA et al. *JAMA Health Forum*.

eTable 6. Health Insurance Type by Income Level, 2021/2022 vs 2019

	Income level	2019 (Pre-pandemic) % (95% CI)	2021 (Pandemic) % (95% CI)	Adjusted relative risk (2021 vs 2019)[a]	*P* value of interaction term[b]	2022 (Pandemic) % (95% CI)	Adjusted relative risk (2022 vs 2019)[a]	*P* value of interaction term[b]
Private	Low	31.4 (30.0, 32.7)	31.0 (29.4, 32.6)	1.00 (0.94, 1.06)	0.04	28.5 (27.0, 29.9)	0.92 (0.86, 0.97)	0.50
	Middle	64.0 (62.7, 65.3)	62.0 (60.7, 63.3)	0.97 (0.95, 1.00)	0.45	60.0 (58.6, 61.3)	0.94 (0.92, 0.97)	0.89
	High	83.2 (82.3, 84.1)	81.6 (80.8, 82.5)	0.98 (0.97, 0.99)	-	80.9 (80.0, 81.8)	0.97 (0.96, 0.98)	-
Medicare	Low	9.7 (9.0, 10.4)	11.8 (11.0, 12.6)	1.13 (1.05, 1.22)	0.62	11.5 (10.6, 12.3)	1.13 (1.04, 1.22)	0.35
	Middle	9.8 (9.1, 10.5)	11.4 (10.6, 12.1)	1.09 (1.01, 1.17)	0.20	12.3 (11.5, 13.0)	1.16 (1.07, 1.25)	0.64
	High	7.1 (6.6, 7.6)	8.6 (8.1, 9.2)	1.17 (1.09, 1.25)	-	8.6 (8.0, 9.1)	1.20 (1.11, 1.28)	-
Medicaid/ Public	Low	30.6 (29.1, 32.1)	31.9 (30.2, 33.5)	1.05 (0.98, 1.11)	0.49	34.7 (33.0, 36.4)	1.14 (1.08, 1.21)	0.64
	Middle	7.6 (6.9, 8.3)	9.5 (8.6, 10.4)	1.25 (1.09, 1.42)	0.56	10.0 (9.1, 10.9)	1.33 (1.16, 1.49)	0.72
	High	1.8 (1.4, 2.1)	2.1 (1.7, 2.4)	1.17 (0.87, 1.46)	-	2.3 (2.0, 2.7)	1.29 (0.99, 1.59)	-
Other[c]	Low	6.3 (5.7, 6.9)	5.9 (5.3, 6.5)	0.91 (0.79, 1.04)	0.42	6.8 (6.1, 7.4)	1.04 (0.91, 1.17)	0.39
	Middle	6.2 (5.5, 6.8)	5.8 (5.3, 6.4)	0.93 (0.80, 1.06)	0.53	6.2 (5.5, 6.8)	0.98 (0.84, 1.11)	0.12
	High	3.9 (3.4, 4.3)	3.8 (3.4, 4.3)	0.98 (0.86, 1.11)	-	4.4 (3.9, 4.9)	1.13 (0.98, 1.28)	-
Uninsured	Low	21.5 (20.1, 22.9)	18.9 (17.6, 20.2)	0.90 (0.83, 0.97)	0.25	18.1 (16.6, 19.5)	0.86 (0.78, 0.93)	0.12
	Middle	12.0 (11.1, 12.9)	10.7 (9.8, 11.6)	0.90 (0.80, 1.00)	0.40	11.1 (10.2, 12.0)	0.93 (0.84, 1.03)	0.78
	High	3.7 (3.2, 4.1)	3.6 (3.2, 4.0)	0.97 (0.81, 1.13)	-	3.5 (3.1, 4.0)	0.95 (0.80, 1.10)	-

[a] Survey-weighted logistic regression models adjusted for participant age and sex.
[b] Models included an interaction term for income-level (low, middle, high) and period (2019 versus 2021/2022), to assess for differential changes in outcomes by income-level during the pandemic. High-income adults were the reference group.
[c] Other includes Children's Health Insurance Program (CHIP), military health insurance, state-sponsored health plans, other government programs, and those who have both Medicare and Medicaid

© 2024 Mein SA et al. *JAMA Health Forum*.

eTable 7. Health Care Use by Income Level, 2021/2022 vs 2019

	**Income level**	**2019 (Pre-pandemic) % (95% CI)**	**2021 (Pandemic) % (95% CI)**	**Adjusted relative risk (2021 vs 2019)[a]**	***P* value of interaction term[b]**	**2022 (Pandemic) % (95% CI)**	**Adjusted relative risk (2022 vs 2019)[a]**	***P* value of interaction term[b]**
Urgent care visits within the last year (% at least one)	Low	27.4 (26.0, 28.8)	24.6 (23.2, 25.9)	0.90 (0.84, 0.96)	0.50	28.7 (27.2, 30.1)	1.05 (0.98, 1.12)	0.003
	Middle	28.3 (27.1, 29.6)	26.2 (25.0, 27.5)	0.93 (0.87, 0.98)	0.91	32.1 (30.7, 33.5)	1.14 (1.07, 1.21)	0.41
	High	30.1 (29.1, 31.2)	27.8 (26.8, 28.9)	0.93 (0.88, 0.97)	-	35.2 (34.0, 36.3)	1.17 (1.11, 1.22)	-
Emergency department visits within the last year (% at least one)	Low	30.4 (29.1, 31.7)	24.8 (23.5, 26.1)	0.81 (0.76, 0.86)	0.10	28.5 (27.2, 29.8)	0.93 (0.88, 0.99)	0.80
	Middle	20.7 (19.7, 21.8)	18.4 (17.4, 19.4)	0.88 (0.82, 0.95)	0.48	19.2 (18.2, 20.2)	0.92 (0.85, 0.99)	0.79
	High	16.0 (15.3, 16.7)	13.5 (12.8, 14.2)	0.85 (0.79, 0.90)	-	14.8 (14.1, 15.6)	0.93 (0.87, 0.99)	-
Hospitalizations within the last year (% yes)	Low	12.3 (11.5, 13.1)	10.9 (10.0, 11.8)	0.87 (0.78, 0.95)	0.52	11.7 (10.9, 12.6)	0.94 (0.85, 1.03)	0.21
	Middle	9.1 (8.4, 9.7)	8.2 (7.6, 8.9)	0.89 (0.80, 0.99)	0.88	8.5 (7.8, 9.2)	0.92 (0.81, 1.02)	0.35
	High	7.3 (6.9, 7.8)	6.6 (6.1, 7.1)	0.90 (0.81, 0.99)	-	6.2 (5.7, 6.7)	0.85 (0.77, 0.94)	-

[a] Survey-weighted logistic regression models adjusted for participant age and sex.
[b] Models included an interaction term for income-level (low, middle, high) and period (2019 versus 2021/2022), to assess for differential changes in outcomes by income-level during the pandemic. High-income adults were the reference group.

© 2024 Mein SA et al. *JAMA Health Forum*.

eTable 8. Health Care and Prescription Medication Affordability by Income Level, 2021 vs 2019 Adjusting for Health Insurance and Health Care Use

	Income level	Age- and sex-adjusted relative risk	Age-, sex-, insurance-adjusted relative risk	Age-, sex-, insurance-, and utilization-adjusted relative risk	*P* value
**Healthcare affordability**					
Delaying care due to cost	Low	0.67 (0.60, 0.74)	0.69 (0.62, 0.77)	0.72 (0.65, 0.80)	<0.001
	Middle	0.85 (0.75, 0.95)	0.88 (0.78, 0.97)	0.91 (0.81, 1.01)	<0.001
	High	1.21 (1.02, 1.40)	1.22 (1.04, 1.40)	1.25 (1.06, 1.43)	
Not seeking care due to cost	Low	0.64 (0.57, 0.72)	0.66 (0.59, 0.74)	0.69 (0.61, 0.78)	<0.001
	Middle	0.82 (0.72, 0.92)	0.85 (0.75, 0.95)	0.89 (0.78, 1.00)	0.03
	High	1.08 (0.88, 1.28)	1.09 (0.90, 1.28)	1.12 (0.92, 1.31)	
Worry about paying medical bills	Low	1.00 (0.96, 1.03)	1.01 (0.97, 1.05)	1.02 (0.98, 1.05)	0.10
	Middle	0.95 (0.92, 0.99)	0.96 (0.93, 1.00)	0.97 (0.94, 1.01)	0.63
	High	0.96 (0.92, 1.00)	0.97 (0.93, 1.01)	0.97 (0.93, 1.01)	
Problems paying medical bills	Low	0.85 (0.78, 0.91)	0.86 (0.79, 0.93)	0.91 (0.84, 0.98)	0.002
	Middle	0.80 (0.73, 0.87)	0.81 (0.74, 0.88)	0.83 (0.76, 0.90)	0.09
	High	0.68 (0.60, 0.76)	0.68 (0.60, 0.77)	0.71 (0.62, 0.79)	
Inability to pay current medical bills[a]	Low	1.01 (0.96, 1.06)	1.02 (0.96, 1.08)	1.03 (0.97, 1.09)	0.77
	Middle	0.96 (0.88, 1.04)	0.96 (0.88, 1.03)	0.96 (0.89, 1.03)	0.18
	High	1.11 (0.94, 1.27)	1.09 (0.94, 1.24)	1.07 (0.93, 1.22)	
**Prescription medication affordability**					
Not filling prescriptions due to cost	Low	0.67 (0.59, 0.74)	0.68 (0.61, 0.76)	0.73 (0.64, 0.81)	0.16
	Middle	0.72 (0.61, 0.82)	0.73 (0.63, 0.83)	0.77 (0.66, 0.88)	0.48
	High	0.79 (0.65, 0.94)	0.80 (0.66, 0.94)	0.82 (0.68, 0.97)	
Delays in filling prescriptions due to cost[b]	Low	0.74 (0.64, 0.84)	0.79 (0.69, 0.89)	0.82 (0.71, 0.93)	0.26
	Middle	0.76 (0.65, 0.87)	0.79 (0.67, 0.90)	0.81 (0.69, 0.92)	0.31
	High	0.69 (0.56, 0.83)	0.69 (0.56, 0.83)	0.70 (0.57, 0.84)	
Skipping doses of medications to save money[b]	Low	0.67 (0.56, 0.77)	0.71 (0.60, 0.82)	0.74 (0.62, 0.85)	0.94
	Middle	0.75 (0.61, 0.88)	0.77 (0.63, 0.91)	0.79 (0.65, 0.93)	0.54
	High	0.69 (0.52, 0.87)	0.70 (0.53, 0.87)	0.71 (0.54, 0.89)	
Taking less medication to save money[b]	Low	0.69 (0.59, 0.79)	0.74 (0.63, 0.84)	0.76 (0.65, 0.87)	0.61
	Middle	0.87 (0.73, 1.02)	0.91 (0.76, 1.05)	0.93 (0.78, 1.08)	0.30
	High	0.78 (0.60, 0.96)	0.78 (0.61, 0.96)	0.80 (0.62, 0.98)	

Survey-weighted logistic regression models that sequentially adjusted for participant age and sex, then insurance status, and then healthcare utilization.
[a] Among individuals who reported having problems paying or being unable to pay any medical bills within the last year.
[b] Among individuals taking prescription medications.

© 2024 Mein SA et al. *JAMA Health Forum*.

eTable 9. Health Care and Prescription Medication Affordability by Income Level, 2022 vs 2019 Adjusting for Health Insurance and Health Care Use

	Income level	Age- and sex-adjusted relative risk	Age-, sex-, insurance-adjusted relative risk	Age-, sex-, insurance-, and utilization-adjusted relative risk	*P* value
**Healthcare affordability**					
Delaying care due to cost	Low	0.73 (0.66, 0.81)	0.77 (0.69, 0.86)	0.79 (0.71, 0.87)	0.008
	Middle	0.84 (0.74, 0.95)	0.87 (0.76, 0.97)	0.88 (0.77, 0.99)	0.17
	High	0.99 (0.82, 1.15)	1.00 (0.84, 1.16)	0.99 (0.84, 1.15)	
Not seeking care due to cost	Low	0.72 (0.64, 0.80)	0.76 (0.68, 0.84)	0.77 (0.69, 0.86)	0.003
	Middle	0.81 (0.71, 0.91)	0.83 (0.73, 0.94)	0.85 (0.74, 0.96)	0.07
	High	1.02 (0.83, 1.21)	1.03 (0.84, 1.22)	1.03 (0.85, 1.22)	
Worry about paying medical bills	Low	0.97 (0.93, 1.00)	0.99 (0.96, 1.03)	0.99 (0.96, 1.03)	0.60
	Middle	0.99 (0.96, 1.03)	1.01 (0.97, 1.04)	1.01 (0.97, 1.04)	0.98
	High	1.00 (0.95, 1.04)	1.00 (0.96, 1.04)	1.00 (0.96, 1.04)	
Problems paying medical bills	Low	0.82 (0.75, 0.88)	0.83 (0.77, 0.90)	0.85 (0.79, 0.92)	0.74
	Middle	0.80 (0.73, 0.88)	0.81 (0.74, 0.89)	0.83 (0.76, 0.91)	0.98
	High	0.80 (0.70, 0.91)	0.81 (0.70, 0.91)	0.80 (0.70, 0.91)	
Inability to pay current medical bills[a]	Low	0.94 (0.89, 1.00)	0.95 (0.89, 1.01)	0.96 (0.90, 1.02)	0.007
	Middle	0.98 (0.90, 1.07)	0.98 (0.90, 1.06)	0.98 (0.90, 1.06)	0.03
	High	1.21 (1.03, 1.38)	1.20 (1.04, 1.36)	1.19 (1.03, 1.35)	
**Prescription medication affordability**					
Not filling prescriptions due to cost	Low	0.75 (0.66, 0.83)	0.77 (0.68, 0.86)	0.79 (0.70, 0.87)	0.08
	Middle	0.84 (0.72, 0.96)	0.85 (0.73, 0.97)	0.87 (0.75, 0.99)	0.49
	High	0.93 (0.77, 1.08)	0.93 (0.78, 1.09)	0.93 (0.77, 1.08)	
Delays in filling prescriptions due to cost[b]	Low	0.74 (0.65, 0.84)	0.78 (0.69, 0.88)	0.79 (0.69, 0.90)	0.07
	Middle	0.76 (0.64, 0.87)	0.79 (0.67, 0.91)	0.79 (0.67, 0.91)	0.06
	High	0.63 (0.50, 0.75)	0.63 (0.50, 0.76)	0.61 (0.49, 0.74)	
Skipping doses of medications to save money[b]	Low	0.67 (0.57, 0.77)	0.71 (0.60, 0.81)	0.72 (0.61, 0.83)	0.35
	Middle	0.68 (0.56, 0.81)	0.71 (0.58, 0.84)	0.72 (0.59, 0.85)	0.34
	High	0.61 (0.45, 0.76)	0.61 (0.46, 0.77)	0.60 (0.45, 0.75)	
Taking less medication to save money[b]	Low	0.65 (0.56, 0.74)	0.69 (0.59, 0.78)	0.70 (0.60, 0.79)	0.84
	Middle	0.70 (0.57, 0.84)	0.73 (0.60, 0.87)	0.74 (0.60, 0.87)	0.51
	High	0.66 (0.51, 0.81)	0.67 (0.51, 0.82)	0.65 (0.50, 0.80)	

Survey-weighted logistic regression models that sequentially adjusted for participant age and sex, then insurance status, and then healthcare utilization.
[a] Among individuals who reported having problems paying or being unable to pay any medical bills within the last year.
[b] Among individuals taking prescription medications.

eTable 10. Health Care Affordability by Income Level Among Adults Aged 18-64 Years, 2021/2022 vs 2019

	Income level	2019 (Pre-pandemic) % (95% CI)	2021 (Pandemic) % (95% CI)	Adjusted relative risk (2021 vs 2019)[a]	*P* value of interaction term[b]	2022 (Pandemic) % (95% CI)	Adjusted relative risk (2022 vs 2019)[a]	*P* value of interaction term[b]
Delaying care due to cost	Low	17.7 (16.5, 18.9)	12.0 (11.0, 13.0)	0.68 (0.60, 0.75)	<0.001	13.1 (12.0, 14.3)	0.74 (0.66, 0.82)	<0.001
	Middle	11.9 (10.9, 12.8)	9.9 (9.0, 10.9)	0.84 (0.74, 0.93)	<0.001	9.9 (8.9, 10.9)	0.84 (0.73, 0.94)	0.05
	High	4.1 (3.6, 4.6)	5.3 (4.8, 5.9)	1.29 (1.08, 1.50)	-	4.2 (3.7, 4.7)	1.02 (0.85, 1.19)	-
Not seeking care due to cost	Low	17.0 (15.7, 18.3)	11.1 (10.0, 12.1)	0.65 (0.57, 0.73)	<0.001	12.5 (11.3, 13.6)	0.73 (0.65, 0.81)	<0.001
	Middle	10.5 (9.6, 11.4)	8.6 (7.7, 9.4)	0.81 (0.71, 0.91)	<0.001	8.4 (7.6, 9.3)	0.80 (0.70, 0.91)	0.02
	High	3.3 (2.8, 3.7)	3.9 (3.4, 4.4)	1.19 (0.97, 1.41)	-	3.5 (3.0, 3.9)	1.06 (0.85, 1.27)	-
Worry about paying medical bills	Low	59.3 (57.5, 61.0)	59.6 (57.8, 61.4)	1.00 (0.96, 1.04)	0.28	57.6 (55.9, 59.4)	0.97 (0.93, 1.01)	0.25
	Middle	56.7 (55.2, 58.2)	54.2 (52.6, 55.8)	0.95 (0.92, 0.99)	0.39	56.6 (54.9, 58.4)	1.00 (0.96, 1.04)	0.94
	High	39.4 (38.1, 40.7)	37.9 (36.6, 39.1)	0.97 (0.92, 1.01)	-	39.3 (38.0, 40.5)	1.00 (0.96, 1.04)	-
Problems paying medical bills	Low	22.0 (20.6, 23.4)	19.0 (17.6, 20.3)	0.86 (0.78, 0.94)	0.01	18.0 (16.7, 19.3)	0.82 (0.75, 0.89)	0.66
	Middle	18.2 (17.0, 19.4)	14.2 (13.2, 15.2)	0.78 (0.70, 0.85)	0.22	14.3 (13.2, 15.4)	0.79 (0.71, 0.87)	0.44
	High	7.1 (6.4, 7.8)	4.7 (4.3, 5.2)	0.68 (0.59, 0.77)	-	5.8 (5.2, 6.4)	0.82 (0.71, 0.94)	-
Inability to pay current medical bills[c]	Low	73.7 (70.8, 76.5)	73.2 (69.8, 76.5)	0.99 (0.94, 1.05)	0.28	69.5 (65.7, 73.2)	0.94 (0.88, 1.00)	<0.001
	Middle	61.7 (58.3, 65.1)	58.8 (54.7, 62.9)	0.95 (0.87, 1.04)	0.10	62.0 (58.0, 66.1)	1.01 (0.92, 1.09)	0.02
	High	40.8 (36.0, 45.6)	45.0 (39.7, 50.4)	1.11 (0.93, 1.29)	-	51.7 (46.5, 56.8)	1.27 (1.07, 1.47)	-

[a] Survey-weighted logistic regression models adjusted for participant age and sex.
[b] Models included an interaction term for income-level (low, middle, high) and period (2019 versus 2021/2022), to assess for differential changes in outcomes by income-level during the pandemic. High-income adults were the reference group.
[c] Among individuals who reported having problems paying or being unable to pay any medical bills within the last year.

© 2024 Mein SA et al. *JAMA Health Forum*.

eTable 11. Prescription Medication Affordability by Income Level Among Adults Aged 18-64 Years, 2021/2022 vs 2019

	Income level	2019 (Pre-pandemic) % (95% CI)	2021 (Pandemic) % (95% CI)	Adjusted relative risk (2021 vs 2019)[a]	*P* value of interaction term[b]	2022 (Pandemic) % (95% CI)	Adjusted relative risk (2022 vs 2019)[a]	*P* value of interaction term[b]
Not filling prescriptions due to cost	Low	13.2 (12.1, 14.3)	8.9 (8.0, 9.8)	0.67 (0.59, 0.76)	0.07	9.9 (8.9, 10.9)	0.75 (0.65, 0.84)	0.04
	Middle	8.2 (7.4, 9.0)	5.7 (5.0, 6.3)	0.69 (0.58, 0.79)	0.18	6.5 (5.7, 7.3)	0.80 (0.68, 0.92)	0.20
	High	3.1 (2.7, 3.5)	2.5 (2.1, 2.8)	0.81 (0.64, 0.97)	-	2.8 (2.4, 3.2)	0.92 (0.75, 1.10)	-
Delays in filling prescriptions due to cost[c]	Low	15.5 (13.9, 17.0)	12.2 (10.7, 13.6)	0.79 (0.67, 0.90)	0.55	11.7 (10.3, 13.0)	0.76 (0.65, 0.86)	0.21
	Middle	11.0 (9.9, 12.2)	8.0 (6.9, 9.1)	0.72 (0.60, 0.84)	0.98	8.0 (7.0, 9.1)	0.73 (0.60, 0.85)	0.28
	High	4.4 (3.8, 5.0)	3.1 (2.5, 3.6)	0.70 (0.55, 0.85)	-	2.7 (2.2, 3.1)	0.61 (0.47, 0.75)	-
Skipping doses of medications to save money[c]	Low	12.2 (10.9, 13.5)	8.6 (7.5, 9.8)	0.71 (0.59, 0.83)	0.89	8.7 (7.5, 9.8)	0.71 (0.59, 0.82)	0.27
	Middle	8.1 (7.2, 9.1)	6.1 (5.2, 7.1)	0.75 (0.60, 0.90)	0.74	5.3 (4.5, 6.2)	0.66 (0.53, 0.79)	0.45
	High	2.8 (2.3, 3.4)	2.0 (1.6, 2.4)	0.70 (0.51, 0.89)	-	1.6 (1.3, 1.9)	0.56 (0.40, 0.72)	-
Taking less medication to save money[c]	Low	13.4 (12.0, 14.8)	9.7 (8.5, 11.0)	0.73 (0.61, 0.84)	0.51	9.1 (7.9, 10.3)	0.68 (0.57, 0.79)	0.83
	Middle	8.4 (7.4, 9.4)	7.2 (6.2, 8.2)	0.86 (0.70, 1.01)	0.59	5.4 (4.5, 6.3)	0.65 (0.51, 0.78)	0.98
	High	3.1 (2.6, 3.6)	2.4 (1.9, 2.9)	0.78 (0.58, 0.98)	-	2.0 (1.6, 2.3)	0.63 (0.46, 0.80)	-

[a] Survey-weighted logistic regression models adjusted for participant age and sex.
[b] Models included an interaction term for income-level (low, middle, high) and period (2019 versus 2021/2022), to assess for differential changes in outcomes by income-level during the pandemic. High-income adults were the reference group.
[c] Among individuals taking prescription medications.

© 2024 Mein SA et al. *JAMA Health Forum*.

eTable 12. Health Care Affordability by Income Level Among Adults Aged ≥65 Years, 2021/2022 vs 2019

	Income level	2019 (Pre-pandemic) % (95% CI)	2021 (Pandemic) % (95% CI)	Adjusted relative risk (2021 vs 2019)[a]	*P* value of interaction term[b]	2022 (Pandemic) % (95% CI)	Adjusted relative risk (2022 vs 2019)[a]	*P* value of interaction term[b]
Delaying care due to cost	Low	6.6 (5.4, 7.8)	4.2 (3.4, 5.0)	0.65 (0.47, 0.83)	0.37	4.7 (3.7, 5.8)	0.71 (0.51, 0.91)	0.89
	Middle	2.7 (2.0, 3.5)	3.2 (2.3, 4.0)	1.14 (0.71, 1.57)	0.01	3.0 (2.1, 3.8)	1.08 (0.67, 1.50)	0.25
	High	1.5 (0.9, 2.1)	0.7 (0.4, 1.0)	0.48 (0.20, 0.75)	-	1.1 (0.6, 1.5)	0.73 (0.33, 1.13)	-
Not seeking care due to cost	Low	6.8 (5.6, 8.0)	4.1 (3.3, 4.9)	0.62 (0.45, 0.79)	0.02	4.6 (3.6, 5.7)	0.68 (0.49, 0.86)	0.72
	Middle	2.4 (1.7, 3.2)	2.7 (2.0, 3.3)	1.08 (0.65, 1.51)	<0.001	2.6 (1.9, 3.4)	1.08 (0.63, 1.53)	0.29
	High	1.5 (0.9, 2.1)	0.4 (0.2, 0.6)	0.27 (0.11, 0.43)	-	1.1 (0.7, 1.5)	0.74 (0.34, 1.14)	-
Worry about paying medical bills	Low	45.5 (43.0, 48.0)	44.1 (41.8, 46.4)	0.97 (0.90, 1.04)	0.93	43.7 (41.3, 46.0)	0.96 (0.89, 1.03)	0.41
	Middle	35.3 (33.1, 37.5)	34.4 (32.2, 36.7)	0.97 (0.89, 1.06)	0.99	35.5 (33.3, 37.6)	1.00 (0.92, 1.09)	0.98
	High	21.4 (19.7, 23.0)	20.5 (18.8, 22.2)	0.97 (0.86, 1.07)	-	21.3 (19.7, 22.9)	1.00 (0.89, 1.11)	-
Problems paying medical bills	Low	17.2 (15.3, 19.1)	13.8 (12.0, 15.6)	0.81 (0.67, 0.94)	0.88	14.0 (12.3, 15.7)	0.81 (0.68, 0.94)	0.69
	Middle	8.9 (7.6, 10.2)	8.6 (7.3, 9.9)	0.96 (0.76, 1.17)	0.25	8.6 (7.2, 10.0)	0.97 (0.77, 1.16)	0.14
	High	3.6 (2.7, 4.4)	2.7 (2.0, 3.4)	0.76 (0.50, 1.01)	-	2.6 (1.9, 3.2)	0.72 (0.48, 0.96)	-
Inability to pay current medical bills[c]	Low	59.6 (53.8, 65.4)	65.5 (59.4, 71.5)	1.10 (0.96, 1.24)	0.61	57.0 (51.0, 63.1)	0.95 (0.82, 1.09)	0.43
	Middle	46.5 (38.6, 54.4)	48.3 (39.7, 56.8)	1.04 (0.78, 1.30)	0.95	42.1 (33.6, 50.6)	0.91 (0.67, 1.15)	0.54
	High	45.3 (33.1, 57.4)	46.5 (33.5, 59.5)	1.02 (0.62, 1.43)	-	35.4 (22.5, 48.4)	0.77 (0.42, 1.11)	-

[a] Survey-weighted logistic regression models adjusted for participant age and sex.
[b] Models included an interaction term for income-level (low, middle, high) and period (2019 versus 2021/2022), to assess for differential changes in outcomes by income-level during the pandemic. High-income adults were the reference group.
[c] Among individuals who reported having problems paying or being unable to pay any medical bills within the last year.

© 2024 Mein SA et al. *JAMA Health Forum*.

eTable 13. Prescription Medication Affordability by Income Level Among Adults Aged ≥65 Years, 2021/2022 vs 2019

	**Income level**	**2019 (Pre-pandemic) % (95% CI)**	**2021 (Pandemic) % (95% CI)**	**Adjusted relative risk (2021 vs 2019)[a]**	***P* value of interaction term[b]**	**2022 (Pandemic) % (95% CI)**	**Adjusted relative risk (2022 vs 2019)[a]**	***P* value of interaction term[b]**
Not filling prescriptions due to cost	Low	7.7 (6.5, 8.9)	5.0 (4.0, 6.0)	0.66 (0.50, 0.83)	0.42	5.9 (4.8, 7.0)	0.76 (0.58, 0.95)	0.18
	Middle	4.0 (3.1, 4.9)	3.8 (2.9, 4.6)	0.93 (0.65, 1.22)	0.51	4.6 (3.7, 5.6)	1.14 (0.80, 1.49)	0.62
	High	2.0 (1.5, 2.5)	1.6 (1.1, 2.0)	0.79 (0.48, 1.09)	-	2.0 (1.4, 2.6)	1.02 (0.65, 1.39)	-
Delays in filling prescriptions due to cost[c]	Low	6.5 (5.3, 7.7)	3.6 (2.7, 4.6)	0.57 (0.39, 0.75)	0.59	4.5 (3.4, 5.5)	0.68 (0.47, 0.88)	0.73
	Middle	4.0 (3.1, 4.8)	4.0 (3.1, 4.9)	1.00 (0.69, 1.30)	0.14	3.9 (3.0, 4.9)	1.00 (0.67, 1.32)	0.28
	High	2.1 (1.5, 2.8)	1.4 (0.9, 1.8)	0.65 (0.35, 0.94)	-	1.6 (1.0, 2.2)	0.74 (0.41, 1.06)	-
Skipping doses of medications to save money[c]	Low	5.2 (4.1, 6.3)	2.6 (1.9, 3.4)	0.52 (0.33, 0.70)	0.40	2.6 (1.9, 3.3)	0.50 (0.33, 0.66)	0.06
	Middle	2.8 (2.1, 3.6)	2.1 (1.4, 2.8)	0.73 (0.42, 1.04)	0.85	2.5 (1.7, 3.4)	0.90 (0.53, 1.27)	0.95
	High	1.3 (0.8, 1.8)	0.8 (0.5, 1.2)	0.67 (0.29, 1.06)	-	1.2 (0.6, 1.7)	0.91 (0.38, 1.45)	-
Taking less medication to save money[c]	Low	6.1 (4.9, 7.3)	3.4 (2.6, 4.2)	0.57 (0.40, 0.74)	0.23	3.3 (2.5, 4.1)	0.53 (0.36, 0.69)	0.08
	Middle	3.3 (2.4, 4.1)	3.2 (2.3, 4.0)	0.97 (0.63, 1.31)	0.55	3.5 (2.6, 4.4)	1.06 (0.66, 1.46)	0.96
	High	1.5 (1.0, 2.1)	1.2 (0.8, 1.6)	0.80 (0.39, 1.21)	-	1.3 (0.8, 1.9)	0.87 (0.45, 1.29)	-

[a] Survey-weighted logistic regression models adjusted for participant age and sex.
[b] Models included an interaction term for income-level (low, middle, high) and period (2019 versus 2021/2022), to assess for differential changes in outcomes by income-level during the pandemic. High-income adults were the reference group.
[c] Among individuals taking prescription medications.

© 2024 Mein SA et al. *JAMA Health Forum*.

eTable 14. Health Care Affordability Among US Adults, 2021/2022 vs 2019

	**2019 (Pre-pandemic) % (95% CI)**	**2021 (Pandemic) % (95% CI)**	**Adjusted relative risk (2021 vs 2019)[a]**	***P* value**	**2022 (Pandemic) % (95% CI)**	**Adjusted relative risk (2022 vs 2019)[a]**	***P* value**
Delaying care due to cost	9.1 (8.6, 9.5)	7.1 (6.7, 7.5)	0.79 (0.73, 0.85)	<0.001	7.0 (6.6, 7.4)	0.78 (0.72, 0.84)	<0.001
Not seeking care due to cost	8.3 (7.8, 8.8)	6.1 (5.7, 6.4)	0.74 (0.68, 0.79)	<0.001	6.3 (5.9, 6.7)	0.76 (0.70, 0.82)	<0.001
Worry about paying medical bills	47.0 (46.1, 47.8)	44.7 (43.9, 45.6)	0.96 (0.93, 0.98)	<0.001	45.5 (44.6, 46.4)	0.97 (0.95, 0.99)	0.02
Problems paying medical bills	13.8 (13.2, 14.3)	10.5 (10.1, 11.0)	0.77 (0.72, 0.81)	<0.001	10.7 (10.3, 11.2)	0.78 (0.74, 0.82)	<0.001
Inability to pay current medical bills[b]	61.8 (59.9, 63.7)	61.8 (59.7, 63.9)	1.00 (0.96, 1.05)	0.85	60.7 (58.4, 62.9)	0.98 (0.94, 1.03)	0.50

[a] Survey-weighted logistic regression models adjusted for participant age and sex.
[b] Among individuals who reported having problems paying or being unable to pay any medical bills within the last year.

© 2024 Mein SA et al. *JAMA Health Forum*.

eTable 15. Prescription Medication Affordability Among US Adults, 2021/2022 vs 2019

	**2019 (Pre-pandemic) % (95% CI)**	**2021 (Pandemic) % (95% CI)**	**Adjusted relative risk (2021 vs 2019)[a]**	***P* value**	**2022 (Pandemic) % (95% CI)**	**Adjusted relative risk (2022 vs 2019)[a]**	***P* value**
Not filling prescriptions due to cost	7.0 (6.6, 7.4)	4.7 (4.4, 5.0)	0.68 (0.62, 0.74)	<0.001	5.4 (5.0, 5.7)	0.78 (0.71, 0.84)	<0.001
Delays in filling prescriptions due to cost[b]	7.9 (7.4, 8.4)	5.5 (5.2, 5.9)	0.71 (0.65, 0.78)	<0.001	5.5 (5.1, 5.8)	0.69 (0.63, 0.76)	<0.001
Skipping doses of medications to save money[b]	5.9 (5.5, 6.3)	3.9 (3.5, 4.2)	0.67 (0.60, 0.75)	<0.001	3.7 (3.4, 4.0)	0.63 (0.56, 0.70)	<0.001
Taking less medication to save money[b]	6.4 (5.9, 6.8)	4.6 (4.3, 5.0)	0.74 (0.66, 0.81)	<0.001	4.1 (3.7, 4.4)	0.64 (0.57, 0.71)	<0.001

[a] Survey-weighted logistic regression models adjusted for participant age and sex.
[b] Among individuals taking prescription medications.

© 2024 Mein SA et al. *JAMA Health Forum*.
