# Supplementary material for: Changes in Health Care and Prescription Medication Affordability in the US During the COVID-19 Pandemic
Source: JAMA Health Forum. 2024 Jun 30;5(6.9):e241939. doi: 10.1001/jamahealthforum.2024.1939 (PMC11215556; doi:10.1001/jamahealthforum.2024.1939)
Supplement: Supplement 2. — Data Sharing Statement [file jamahealthforum-e241939-s002.pdf]

Data Sharing Statement

Mein. Changes in Health Care and Prescription Medication Affordability in the US During the COVID-19 Pandemic. *JAMA Health Forum*. Published June 30, 2024.
doi:10.1001/jamahealthforum.2024.1939

Data
Data available: No

Additional Information
Explanation for why data not available: We used publicly available data for this study.
